# Mononuclear Nickel(II) Complexes with Schiff Base Ligands: Synthesis, Characterization, and Catalytic Activity in Norbornene Polymerization

**DOI:** 10.3390/polym9030105

**Published:** 2017-03-16

**Authors:** Yi-Mei Xu, Kuan Li, Yuhong Wang, Wei Deng, Zi-Jian Yao

**Affiliations:** School of Chemical and Environmental Engineering, Shanghai Institute of Technology, Shanghai 201418, China; 166061228@sit.edu.cn (Y.-M.X.); 156061209@sit.edu.cn (K.L.); yuhong_wang502@sit.edu.cn (Y.W.)

**Keywords:** Schiff base, X-ray crystals, catalysis, polymerization, nickel(II) complex

## Abstract

The nickel(II) catalyst has manifested higher catalytic activity compared to that of other late transition metal catalysts for norbornene polymerization. Therefore, several structurally similar *trans*-nickel(II) compounds of *N*,*O*-chelate bidentate ligands were synthesized and characterized. Both the electronic effect and the steric hindrance influence polymerization. The molecular structures of **2**, **4** and **5** were further confirmed by single-crystal X-ray diffraction.

## 1. Introduction

**Scheme 1:**
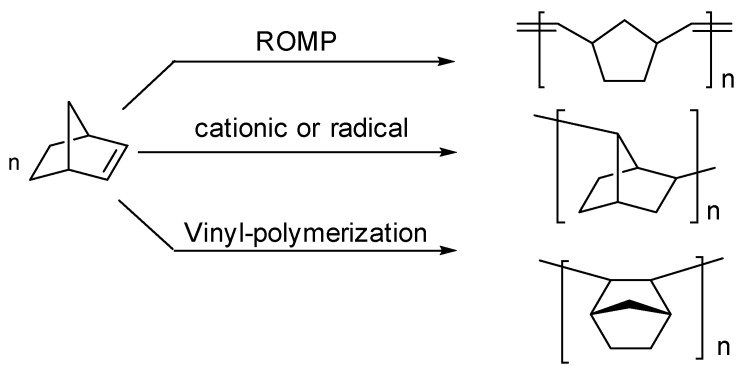
Three different types of polymerization for norbornene.

Over the past two decades, polynorbornene (PNB) has been widely utilized in industrial production because of its excellent physical and chemical properties, including its high solubility in ordinary organic solvents, its excellent heat resistivity, and its optical transparency [1,2,3,4]. There are three pathways for norbornene polymerziation (Scheme 1): (i) Ring-opening metathesis polymerization (ROMP). The obtained product still contains double bonds in the backbone of polynorbornene [5]. (ii) Cationic or radical polymerization. Polynorbornenes synthesized by this route often show low molecular weights [6]. (iii) Vinyl-type polymerization. The bicyclic motifs remain in the polymer chain of the polynorbornene given via this polymerization route [7]. It was obtained first by using a TiCl_4_-based Ziegler catalyst, but these and subsequent zirconocene/MAO catalytic systems perform with lower activities [8,9,10]. The products (PNB) displays a characteristically rigid random coil conformation, which shows a restricted rotation around the main chain and exhibits high thermal stability (*T*_g_ > 350 °C).

Under the stimulation of academic and commercial factors, different transition metal complexes based on various ligands, such as bidentate ligands N^N [11,12], N^O [13,14,15,16,17], N^P [18], and P^P [19] and tridentate ligands N^N^N [20], N^N^O [21], and N^P^N [22], were designed and prepared for olefin polymerization. Among these metal complexes, Ni-based catalysts are best known to oligomerize ethylene and dimerize propylene and higher R-olefins because, before 1995, nickel(II) metal was generally thought to prefer α-hydride elimination followed by reductive elimination [23,24,25,26]. However, transition-metal complexes with ligands containing dissimilar donor atoms have been widely studied, primarily for their applications in important homogeneous catalytic processes [27,28,29]. In particular, the complexes bearing bidentate N^O ligands have drawn more attention than ever before. Additionally, on the basis of previous reports, nickel(II) complexes with N^O ligands often exhibited good catalytic activity for olefin polymerization [13,14,15].

We are always interested in studying novel intramolecularly coordinated nickel(II) complexes with polymerization activity. Here, a series of mononuclear nickel(II) complexes based on the *bis*-N^O-chelate ligand was designed and synthesized so that their polymerization activity could be investigated. These complexes exhibited catalytic activity that is good for the polymerization of norbornene in the presence of methylaluminoxane (MAO) as a co-catalyst. Moreover, the effects that influenced catalytic behavior are herein discussed.

## 2. Results and Discussion

### 2.1. Synthesis of N,O Bidentate Ligands **HL1**–**HL5**

**Scheme 2:**
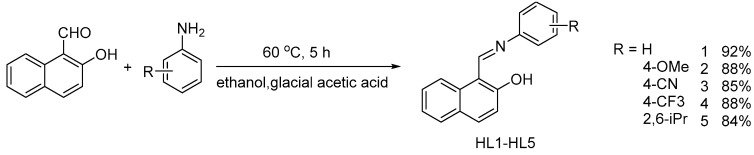
Synthesis of *N*,*O*-chelate bidentate ligands **HL1**–**HL5**.

According to previous methods [30,31,32], the bidentate ligands **HL1**–**HL5** were obtained in high yields by the reaction between 2-hydroxy-1-naphthaldehyde and corresponding aryl amines in ethanol solutions. They were isolated as yellow solids in high yields (Scheme 2). The ^1^H-NMR spectra and elemental analysis data all ascertain the identity of the ligands.

### 2.2. Synthesis of N,O-Coordinate Ni(II) Complexes **1**–**5**

**Scheme 3:**
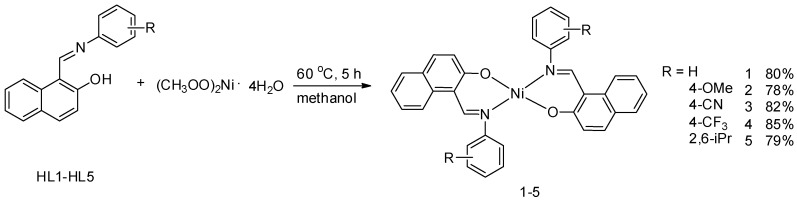
Synthesis of *N*,*O*-coordinate Ni(II) Complexes **1**–**5**.

Analogous to the procedure of preparing copper(II) complexes [13,14,15], the reactions of Schiff base ligands **HL1**‒**HL5** with nickel(II) acetate yielded the *N*,*O*-chelate Ni(II) Complexes **1**–**5** in good yields (Scheme 3). The products precipitated from the solution after the reaction was cooled to room temperature. Complexes **1**–**5** are soluble in toluene and dichloromethane but slightly soluble in diethyl ether and petroleum ether. They are stable at room temperature in air. No decomposition was observed even after refluxing in toluene for several hours. HRMS spectra of complexes **1**–**5** have exhibited strong molecular peaks (Figures S1–S5). Meanwhile, TGA characterization confirmed their thermal stability (Figure S6). However, the ^1^H and ^13^C-NMR spectra of these complexes are not informative because of the *para*-magnetism of the nickel(II) complexes. The IR spectra of *N*,*O*-chelate Ni(II) complexes are similar to each other. The common feature of these complexes is that the C=N stretching vibration of the ligands (1625–1635 cm^−1^) are shifted to lower frequencies (1610 cm^−1^).

### 2.3. UV–Vis Spectroscopy

**Figure 1:**
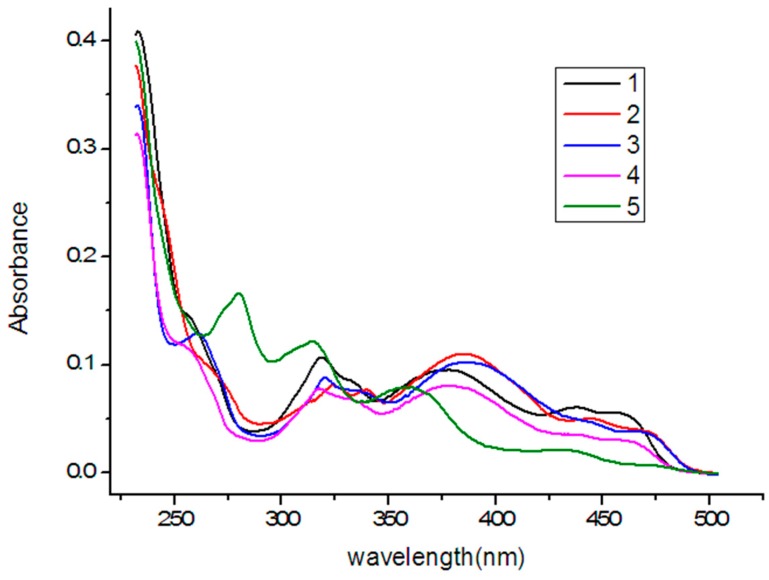
UV–Vis spectra of Complexes **1**–**5** (3.59 × 10^−6^ mol·dm^−3^ in CH_2_Cl_2_).

Figure 1 shows the UV–Vis spectra of the nickel(II) Complexes **1**–**5** in dichloromethane solutions. The UV–Vis absorption spectral data is presented in Table 1. The absorption spectra of all complexes is characterized by intense absorption bands in the range of 319–324 nm, which are assigned to the **π**→π* molecular orbitals localized on the imine group and the aromatic ring [33]. The lower-intensity absorption bands in the 366–382 nm region are assigned to the metal-to-ligand charge transfer (MLCT) [34]. The ultraviolet spectra measurements of these complexes demonstrate that their electronic structures are similar to each other.

### 2.4. X-ray Crystallographic

**Figure 2:**
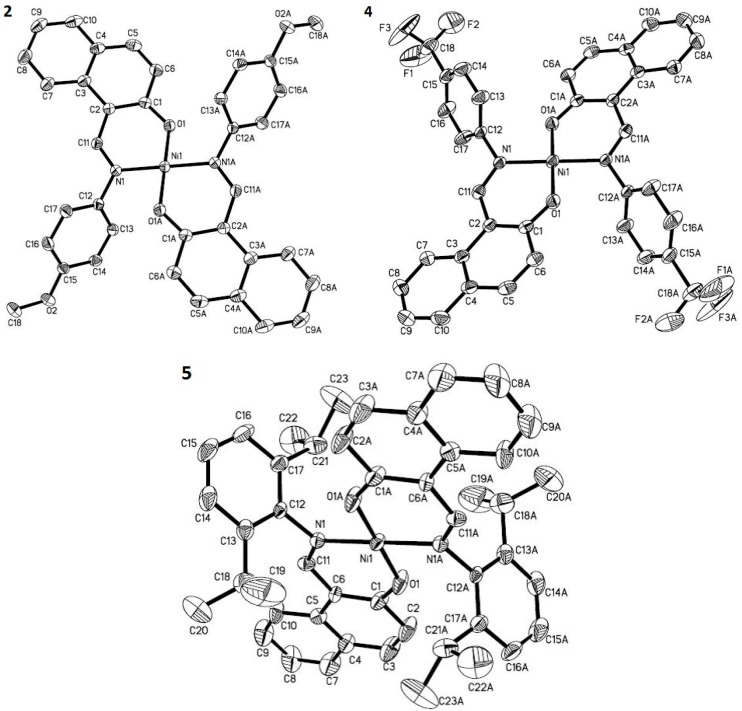
Molecular structures of **2**, **4,** and **5** with 30% probability ellipsoids. Hydrogen atoms are omitted for clarity.

In order to identify the structures of these nickel(II) complexes, single-crystal X-ray diffraction measurement was carried out on Complexes **2**, **4**, and **5**. Suitable crystals were obtained by slow diffusion of n-hexane into their concentrated solution of dichloromethane. Selected bond lengths and angles (Table S1) and their crystal data (Table S2) are listed in the supporting information. As shown in Figure 2, they have analogous structures in a solid state. Their structures are centrosymmetric with the symmetry centers located on the metal centers. Although previous reports indicated that the steric and electron effects of the substituent groups on the aromatic rings of bulky four-coordinated transition metal complexes probably distort the geometry of the metal center from the planar coordination to the *pseudo*-tetrahedral geometry [35,36,37]; however, the NiN_2_O_2_ chromophores of the three nickel(II) complexes are absolutely planar with the dihedral angles of 0 between the planes of N(1)–Ni(1)–O(1) and N(1A)–Ni(1)–O(1A). The Ni(1)–N(1) and Ni(1)–O(1) distances are both located in the range of known values for these bonds in analogous complexes [38]. The structures of the three complexes are similar to each other except for the slight differences in bond distance and angles. We failed to obtain single crystals of other nickel(II) complexes after many attempts.

### 2.5. Polymerization of Norbornene

**Scheme 4:**
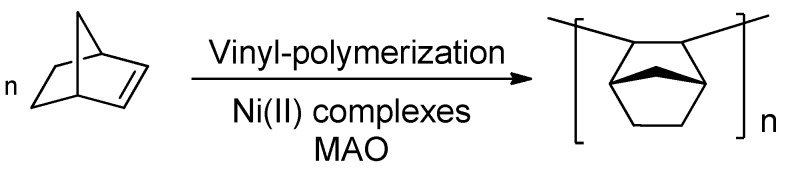
Vinyl-polymerization catalyzed by Ni(II) complexes.

The catalytic behavior of these nickel(II) complexes was investigated based on our previous work [39]. Preliminary experiments on norbornene polymerization were carried out in the presence of MAO as a co-catalyst. We chose chlorobenzene as the reaction solvent for the polymerization process because the polar solvent was able to improve the catalytic performances in norbornene vinyl polymerization (Scheme 4). Experimental results are summarized in Table 2. No catalytic activity was observed for Complex **1** without the addition of MAO (Table 2, Entry 1). For Complex **1**/MAO catalytic system, the optimal molar ratio of Al/Ni was 2500 (Table 2, Entries 6 and 8). The catalytic activity and molecular weight (*M*_w_) of the polymer were higher in the presence of increasing amounts of MAO (Table 2, Entries 2–6). However, the catalytic activity slightly decreased when the Al/Ni ratio was increased to 3000 (Table 2, Entry 7). The catalyst performance was also influenced by the reaction temperature, and 60 °C is the best choice (Table 2, Entry 8). The decrease of catalytic activity was observed at higher temperatures (90 °C), probably because of the decomposition or instability of the active species formed by the catalytic precursors (Table 2, Entry 9). For Complex **4**, the highest catalytic activity, up to 3.06 × 10^6^ gPNB mol^−1^ Ni h^−1^, was obtained at 60 °C in the optimal Al/Ni molar ratio (Table 2, Entry 12). This complex showed much higher catalytic activity (almost two times) than that of the *C*,*S*-chelate nickel(II) complex based on the carborane ligand [38]. In comparison with the nickel complex(II) with N,P-ligand [7], the catalytic activity of Complex **4** is lower; however, a substantial amount of MAO should be used in the polymerization by using *N*,*P*-chelate nickel(II) complex as a catalyst, and the molecular weight of the obtained polynorbornenes is very low (6.47 × 10^5^ g·mol^−1^). Results showed that both electronic and steric effects of the substituted groups had an influence on the catalyst performance. Generally, the transition metal complexes with an electron-withdrawing group that bonded to the ligands often showed higher catalytic activity, because the electron-withdrawing group made the metal center more electron-deficient and the olefin could easily coordinate to the metal center [39]. On the other hand, bulky substituted groups bonded to the transition metal complexes are of great benefit to the polymerization, because the bulky groups can protect the metal center and β-H elimination is inhibited in the polymerization process. For example, Complex **4** with the 4-trifluoromethyl group exhibited higher catalytic activity than that of Complexes **1**, **2**, **3**, and **5** (Table 2, Entries 8 and 10–13). This result indicates that the electron effects play a more important role than that of the steric effects for these nickel(II) complexes.

The obtained polynorbornenes were characterized by IR and-NMR spectroscopy (Figure S7). The characteristic peaks of these polymers at approximately 942 cm^−1^ were analogous. These absorption peaks at about 942 cm^−1^ were identified to the ring system of bicycle heptane [40]. There are no absorptions at about 735 and 960 cm^−1^, suggesting that no ROMP structure of polynorbornenes were found, because the two absorptions are assign to the *trans* and *cis* forms of double bonds, respectively, which are characteristic of the ROMP structure of polynorbornenes [41,42,43]. The ^1^H HMR polynorbornenes displayed four groups of signals in the range of δ 1.0–3.0 ppm. The absence of the resonances at approximately δ 5.1 and 5.3 ppm in the ^1^H-NMR spectra further confirms that the polymers are vinyl-type addition products [44].

## 3. Experimental Section

### 3.1. General Data 

All experiments were carried out under an atmosphere of nitrogen using standard Schlenk techniques. Ethanol and methanol were used as commercial products without further purification. ^1^H-NMR (500 MHz) spectra was measured with a Bruker DMX‒500 spectrometer (Bruker, Washington, DC, USA). Elemental analysis was performed on an Elementar vario EL III analyzer (Elementar, Langenselbold, Germany). UV–Vis absorption spectra were recorded using a UV 765 spectrophotometer (Shimadzu, Kyoto, Japan) with quartz cuvettes with a 1 cm path length. IR (KBr) spectra were measured with the Nicolet FT-IR spectrophotometer (Thermo Fisher Scientific, Waltham, MA, USA).

### 3.2. Synthesis of Ligands **HL1**–**HL5**

The Schiff base ligands **HL1**–**HL5** were synthesized by a routine method. 2-Hydroxy-1-naphthaldehyde (5.0 mmol) and corresponding amines (5.0 mmol) were combined and heated to reflux in ethanol for 6 h in the presence of catalytic amount of ethyl acetate, resulting in a color change from colorless to bright yellow. Solvent was concentrated under reduced pressure and stored at −10 °C overnight, and further purification was achieved by filtration and washed with CH_3_OH (3 × 10 mL). The collected solid was dried under vacuum.

**HL1**: yellow solid; 1.13 g, 92% yield. ^1^H-NMR (500 MHz, CDCl_3_, 25 °C): δ 15.54 (s, 1H), 9.36 (d, *J* = 4.5 Hz, 1H), 8.13 (d, *J* = 8.5 Hz, 1H), 7.83 (d, *J* = 9.5 Hz, 1H), 7.74 (d, *J* = 8.0 Hz, 1H), 7.56 (t, *J* = 9.0 Hz, 1H), 7.51 (t, *J* = 9.0 Hz, 2H), 7.41–7.30 (m, 5H). IR (KBr, disk): *υ* 3066, 1621, 1568, 1488, 1331, 821, 750, 695 cm^−1^. Elemental analysis calcd (%) for C_17_H_13_NO: C 82.57, H 5.30, N 5.66, found: C 82.68, H 5.35, N 5.47.

**HL2**: yellow solid; 1.22 g, 88% yield. ^1^H-NMR (500 MHz, CDCl_3_, 25 °C): δ 15.71 (s, 1H), 9.35 (d, *J* = 4.0 Hz, 1H), 8.14 (d, *J* = 8.5 Hz, 1H), 7.82 (d, *J* = 9.0 Hz, 1H), 7.75 (d, *J* = 8.0 Hz, 1H), 7.56 (t, *J* = 9.6 Hz, 1H), 7.38–7.34 (m, 3H), 7.14 (d, *J* = 9.0 Hz, 1H), 7.02 (d, *J* = 9.0 Hz, 2H), 3.88 (s, 3H). IR (KBr, disk): *υ* 3051, 1621, 1507, 1302, 1252, 821, 750, 502 cm^−1^. Elemental analysis calcd (%) for C_18_H_15_NO_2_: C 77.96, H 5.45, N 5.05, found: C 77.79, H 5.38, N 5.23.

**HL3**: yellow solid; 1.41 g, 85% yield. ^1^H-NMR (500 MHz, CDCl_3_, 25 °C): δ 15.23 (s, 1H), 9.08 (d, *J* = 3.0 Hz, 1H), 8.00 (d, *J* = 8.5 Hz, 1H), 7.88 (d, *J* = 9.0 Hz, 1H), 7.78 (d, *J* = 8.0 Hz, 1H), 7.52 (t, *J* = 8.0 Hz, 1H), 7.37 (t, *J* = 7.5 Hz, 1H), 7.26–7.25 (m, 3H), 7.21 (d, *J* = 9.5 Hz, 1H), 3.16–3.07 (m, 2H), 1.24 (d, *J* = 7.0 Hz, 12H). IR (KBr, disk): *υ* 3062, 1624, 1463, 1330, 1246, 824, 796, 747 cm^−1^. Elemental analysis calcd (%) for C_23_H_25_NO: C 83.34, H 7.60, N 4.23, found: C 83.39, H 7.53, N 4.36.

**HL4**: yellow solid; 1.38 g, 88% yield. ^1^H-NMR (500 MHz, CDCl_3_, 25 °C): δ 15.12 (s, 1H), 9.40 (d, *J* = 2.5 Hz, 1H), 8.15 (d, *J* = 8.5 Hz, 1H), 7.88 (d, *J* = 9.5 Hz, 1H), 7.76–7.73 (m, 3H), 7.59 (t, *J* = 7.5 Hz, 1H), 7.46 (d, *J* = 8.5 Hz, 2H), 7.41 (t, *J* = 7.5 Hz, 1H), 7.15 (d, *J* = 9.0 Hz, 1H). IR (KBr, disk): *υ* 3057, 1627, 1584, 1320, 1156, 753, 595 cm^−1^. Elemental analysis calcd (%) for C_18_H_12_F_3_NO: C 68.57, H 3.84, N 4.44, found: C 68.63, H 3.85, N 4.55.

**HL5**: yellow solid; 1.14 g, 84% yield. ^1^H-NMR (500 MHz, CDCl_3_, 25 °C): δ 14.94 (s, 1H), 9.40 (d, *J* = 3.0 Hz, 1H), 8.15 (d, *J* = 8.5 Hz, 1H), 7.90 (d, *J* = 9.5 Hz, 1H), 7.79 (d, *J* = 8.5 Hz, 3H), 7.60 (t, *J* = 7.5 Hz, 1H), 7.46–7.40 (m, 3H), 7.16 (d, *J* = 9.0 Hz, 1H). IR (KBr, disk): *υ* 3060, 1624, 1586, 1308, 1156, 953, 829, 754 cm^−1^. Elemental analysis calcd (%) for C_18_H_12_N_2_O: C 79.39, H 4.44, N 10.29, found: C 79.45, H 4.49, N 10.18.

### 3.3. Synthesis of Nickel(II) Complexes **1**–**5**

A mixture of acetic acid nickel(II) salt Ni(OAc)_2_**^.^**H_2_O (0.15 mmol) and methanol (10 mL) was added to the solution of the ligands **HL1**–**HL5** (0.3 mmol) in methanol (10 mL) [15]. After stirring at 60 °C for 5 h, the deep green solid was collected by filtration and washed with methanol for several times.

**1**: deep green soild; 595 mg, 72% yield. IR (KBr, disk): *υ* 1612, 1574., 1530, 751.71, 697 cm^−1^. Elemental analysis calcd (%) for C_34_H_24_N_2_NiO_2_: C 74.08, H 4.39, N 5.08; Found: C 74.00, H 4.37, N 5.11. ESI-HRMS: *m*/*z* calcd for C_34_H_24_N_2_NiO_2_ [M + H]^+^: 551.1270; Found: 551.1261.

**2** deep green soild; 713 mg, 78% yield. IR (KBr, disk): *υ* 1615, 1598, 1536, 1500, 1356, 1238, 1185, 822, 748 cm^−1^. Elemental analysis calcd (%) for C_36_H_28_N_2_NiO_4_: C 70.73, H 4.62, N 4.58; Found: C 70.79, H 4.52, N 4.57. ESI-HRMS: *m*/*z* calcd for C_36_H_28_N_2_NiO_4_ [M + H]^+^: 611.1481; Found: 611.1495.

**3**: brown soild; 612 mg, 68% yield. IR (KBr, disk): *υ* 1598, 1530, 1497, 1433, 1364, 1190, 827, 746 cm^−1^. Elemental analysis calcd (%) for C_36_H_22_N_4_NiO_2_: C 71.91, H 3.69, N 9.32; Found: C 71.96, H 3.67, N 9.31. ESI-HRMS: *m*/*z* calcd for C_36_H_22_N_4_NiO_2_ [M + H]^+^: 601.1174; Found: 601.1172.

**4**: brown soild; 761 mg, 74% yield. IR (KBr, disk): *υ* 1616, 1602, 1580, 1536, 1454., 1326, 1123, 832, 747 cm^−1^. Elemental analysis calcd (%) for C_36_H_22_F_6_N_2_NiO_2_: C 62.92, H 3.23, N 4.08; Found: C 62.96, H 3.27, N 4.02. ESI-HRMS: *m*/*z* calcd for C_36_H_22_F_6_N_2_NiO_2_ [M + H]^+^: 687.1017; Found: 687.1030.

**5**: brown soild; 776 mg, 72% yield. IR (KBr, disk): *υ* 1615, 1603, 1580, 1537, 1364, 827, 741 cm^−1^. Elemental analysis calcd (%) for C_46_H_48_N_2_NiO_2_: C 76.78, H 6.72, N 3.89; Found: C 76.78, H 6.77, N 3.86. ESI-HRMS: *m*/*z* calcd for C_46_H_48_N_2_NiO_2_ [M + H]^+^: 719.3148; Found: 719.3135.

### 3.4. Norbornene Polymerization 

In a typical procedure, 1 μmol of nickel(II) Complex **1** in 1.0 mL of chlorobenzene, 1.80 g of norbornene in 3 mL of chlorobenzene and 6 mL of fresh chlorobenzene were added to a special polymerization bottle (50 mL) under a nitrogen atmosphere. After stirring at 30 °C for 10 min, a certain amount of MAO was charged into the polymerization system via a syringe and the reaction was started. After 30 min, acidic ethanol (*V*_ethanol_:*V*_conc.HCl_ = 20:1) was added to terminate the reaction. The PNB was isolated by filtration, washed with ethanol, and dried at 80 °C for 24 h under vacuum. For all polymerization procedures, the total reaction volume was 10.0 mL, which could be achieved by varying the amount of chlorobenzene when necessary. The viscosity average molar masses (*M_v_*) of the PNB were obtained in chlorobenzene at 25 °C using Mark–Houwink coefficients.

### 3.5. X-ray Crystallography

Data of **2, 4**, and **5** were collected on a Bruker Smart APEX CCD diffractometer with graphite-monochromated Mo*Kα* radiation (λ = 0.71073 Å). All data were collected at room temperature, and the structures were solved by direct methods and subsequently refined on *F*^2^ by using full-matrix least-squares techniques (SHELXL) [45]. SADABS [46] absorption corrections were applied to the data, all non-hydrogen atoms were refined anisotropically, and hydrogen atoms were located at calculated positions. All calculations were performed using the Bruker program Smart.

## 4. Conclusions

In this report, a series of *N*,*O*-chelate Schiff base Ni(II) complexes containing 2-hydroxy-1-naphthaldehyde ligands was synthesized and characterized by IR spectra, UV, ^1^H-NMR, and single-crystal X-ray diffraction analysis. Structural analysis of **2**, **4**, and **5** confirms that the nickel(II) atom coordinates with two ligands. The results of this experiment demonstrate that these nickel(II) complexes have extremely high catalytic activity (3.06 × 10^6^ gPNB mol^−1^ Ni h^−1^) for norbornene polymerization when using MAO as a co-catalyst. The utilization of these nickel(II) complexes as catalysts in the oxidation of olefins is currently underway in our laboratory.

## Figures and Tables

**Table 1:** UV–Vis Spectra Data of Complexes **1**–**5** in CH_2_Cl_2_.

Complex	λ_abs_/nm (ε/dm^3^·mol^−1^·cm^−1^)
1	319 (2.97 × 10^4^); 378 (2.67 × 10^4^)
2	324 (2.28 × 10^4^); 382 (3.06 × 10^4^)
3	320 (2.45 × 10^4^); 382 (2.24 × 10^4^)
4	319 (2.17 × 10^4^); 377 (2.26 × 10^4^)
5	316 (3.40 × 10^4^); 366 (2.14 × 10^4^)

**Table 2:** Polymerization of norbornene Complexes **1**–**5** activated by MAO *^a^*.

Entry	Catalyst	Al/Ni	*T*/°C	Yield/g	Activity *^b^*	*M*_v_ *^c^*
1	1	0	30	0	–	–
2	1	500	30	0.43	0.86	1.21
3	1	1000	30	0.51	1.02	1.44
4	1	1500	30	0.59	1.18	1.63
5	1	2000	30	0.92	1.84	1.96
6	1	2500	30	1.08	2.16	2.09
7	1	3000	30	0.96	1.92	1.88
8	1	2500	60	1.23	2.46	2.03
9	1	2500	90	0.65	1.30	2.01
10	2	2500	60	1.33	2.35	2.23
11	3	2500	60	1.29	2.58	2.66
12	4	2500	60	1.53	3.06	2.85
13	5	2500	60	1.42	2.84	2.37

*^a^* Polymerization conditions: [NB] = 1.80 g; *V*_total_ = 10 mL; catalyst: nickel Complexes **1**–**5**, 1 μmol, reaction time: 30 min, solvent: chlorobenzene. *^b^* 10^6^ gPNB mol^−1^ Ni h^−1^. *^c^* 10^6^ g·mol^−1^, measured in chlorobenzene at 25 °C using the Mark–Houwink coefficients.

## Supplementary Materials

The following are available online at www.mdpi.com/2073-4360/9/3/105/s1, Figure S1: HRMS of complex **1**. Figure S2: HRMS of complex **2**. Figure S3: HRMS of complex **3**. Figure S4: HRMS of complex **4**. Figure S5: HRMS of complex **5**. Figure S6: TGA curves of complexes **1**‒**5**. Figure S7: IR spectrum and ^1^H NMR spectrum of PNB. Table S1: Selected Bond Lengths (Å) and Angles (◦) for Complexes **2**, **4**, and **5**. Table S2: Crystal data for Complexes **2**, **4**, and **5**.

## Abbreviations

**PNB**	polynorbornenes
**MAO**	methylaluminoxane
