# Supplementary material for: Mononuclear Nickel(II) Complexes with Schiff Base Ligands: Synthesis, Characterization, and Catalytic Activity in Norbornene Polymerization
Source: Polymers (Basel). 2017 Mar 16;9(3):105. doi: 10.3390/polym9030105 (PMC6432298; doi:10.3390/polym9030105)
Supplement: Supplementary file 1 [file polymers-09-00105-s001.pdf]

Supporting Information: Mononuclear Nickel(II) Complexes with Schiff Base Ligands: Synthesis, Characterization, and Catalytic Activity in Norbornene Polymerization

Xu Yi-Mei, Kuan Li, Yuhong Wang and Wei Deng and Zi-Jian Yao

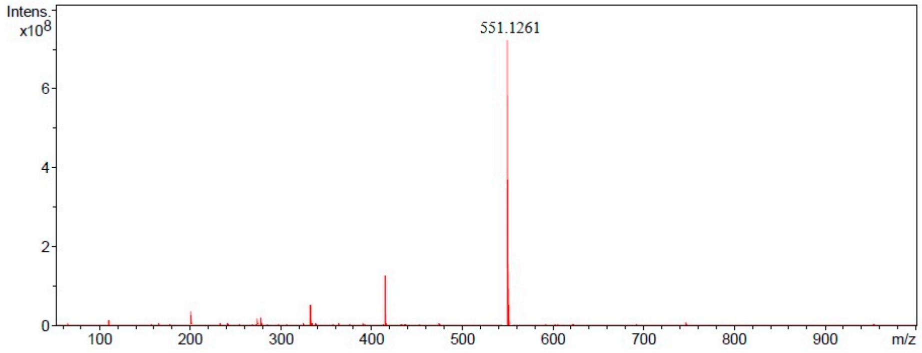

Figure S1. HRMS of complex **1**.

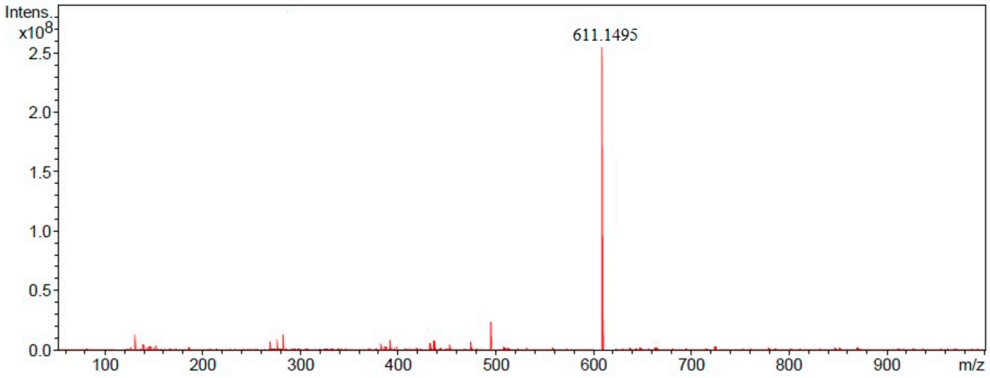

Figure S2. HRMS of complex **2**.

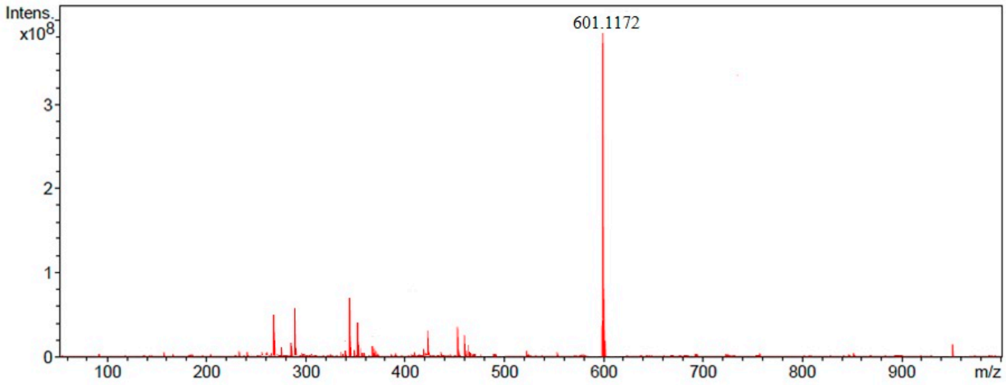

Figure S3. HRMS of complex **3**.

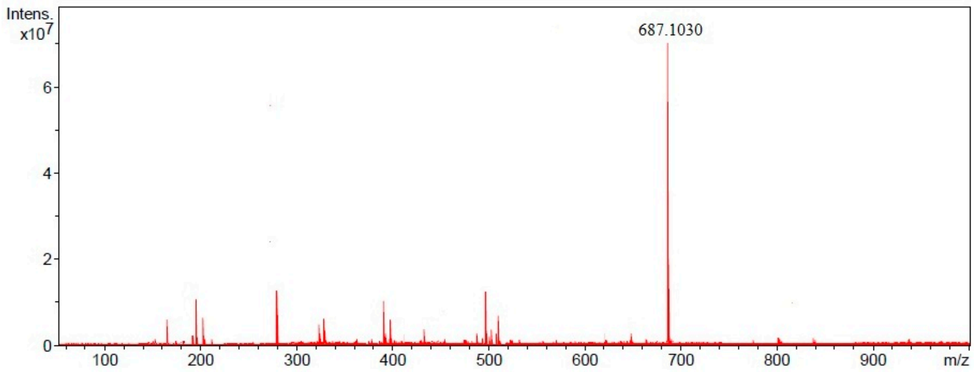

Figure S4. HRMS of complex **4**.

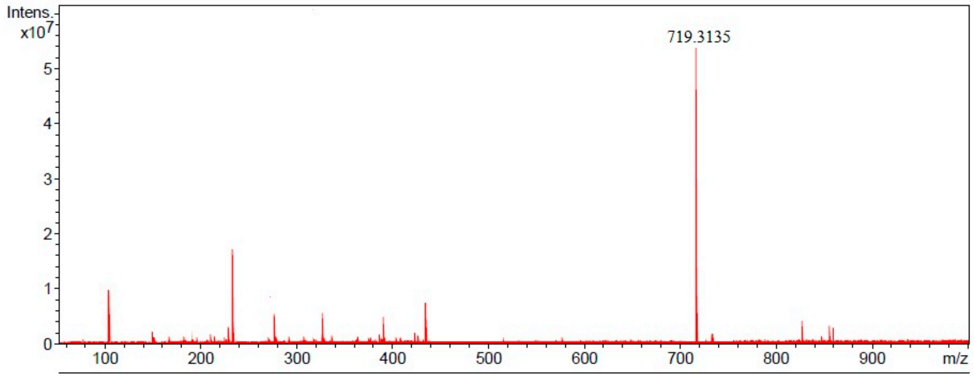

Figure S5. HRMS of complex **5**.

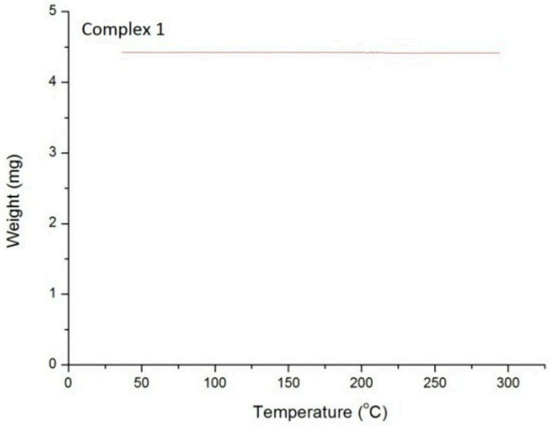

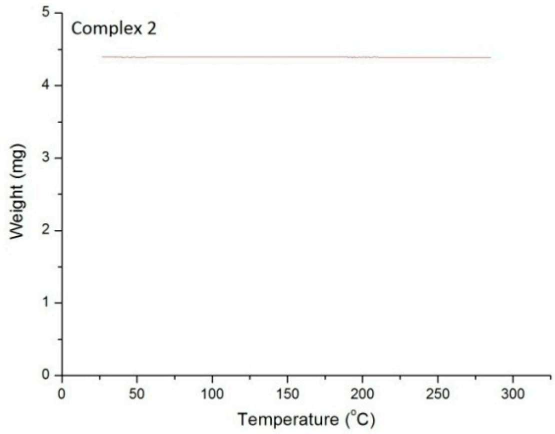

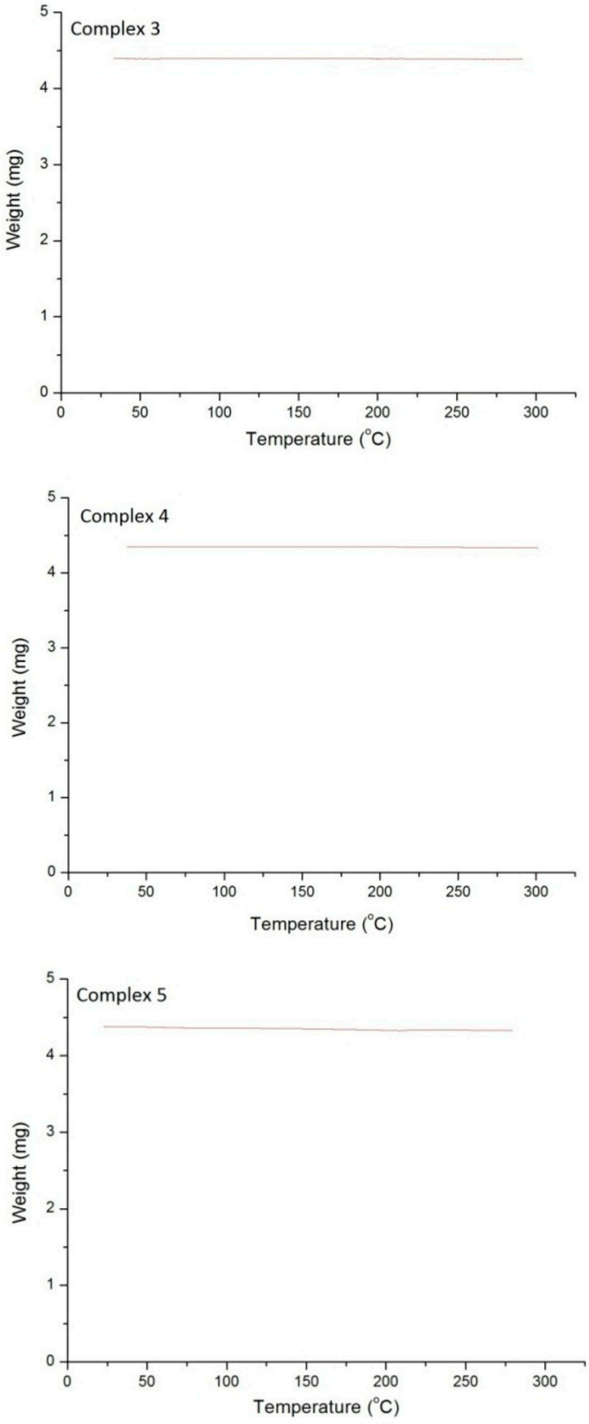

Figure S6. TGA curves of complexes **1–5**.

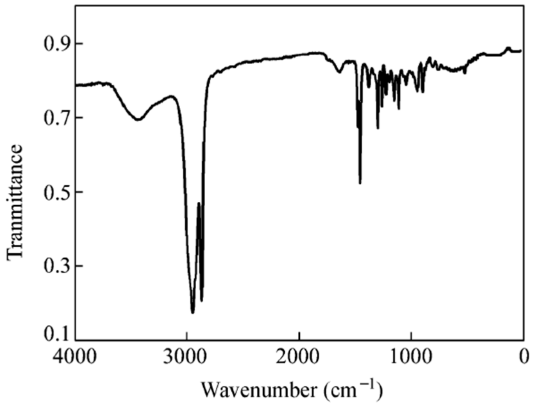

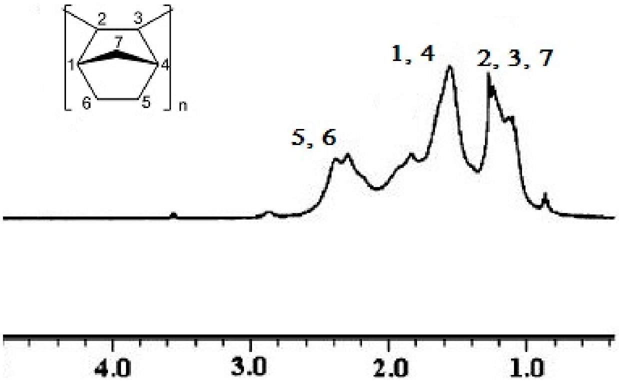

Figure S7. IR spectrum and ^{1}H NMR spectrum of PNB.

Table S1. Selected Bond Lengths (Å) and Angles (◦) for Complexes **2, 4**, and **5**.

Bond lengths (Å) and angles	2	4	5
Ni(1)–N(1)	1.899(3)	1.901(3)	1.906(4)
Ni (1)–N(1A)	1.899(3)	1.901(3)	1.906(4)
Ni(1)–O(1)	1.833(2)	1.832(2)	1.819(4)
Ni(1) –O(1A)	1.833(2)	1.832(2)	1.819(4)
N(1)–C(11)	1.300(4)	1.310(5)	1.301(6)
O(1)–C(1)	1.308(4)	1.295(4)	1.298(6)
O(1)–Ni(1)–N(1)	88.25(11)	87.44(11)	87.76(16)
O(1)–Ni(1)–N(1A)	91.75(11)	92.56(11)	92.24(16)
O(1A)–Ni(1)–N(1)	89.69(6)	92.56(11)	92.24(16)
O(1A)–Ni(1)–N(1A)	90.31(6)	87.44(11)	87.76(16)
N(1)–Ni(1)–N(1A)	180.00(16)	180.00(2)	180.00(2)
O(1)–Ni(1)–O(1A).	180.00(9)	180.00(2)	180.00(1)

Table S2. Crystal data for Complexes **2, 4**, and **5**[a].

	**2**	**4**	**5**
	**2**	**4**	**5**
Formula	$C_{36}H_{28}N_2NiO_4$	$C_{36}H_{22}F_6N_2NiO_2$	$C_{46}H_{48}N_2NiO_2$
Fw	611.31	687.27	719.57
T/K	293(2)	293(2)	293(2)
Crystal system	monoclinic	monoclinic	triclinic
Space group	*P*2₁/c	*C*2/*c*	*P*-1
a/ Å	10.5790(4)	24.966(3)	9.3981(7)
b/ Å	9.6679(3)	5.8753(6)	9.7509(13)
c/ Å	14.3723(6)	21.035(3)	11.4639(15)
α/°	90.00	90.00	80.721(11)
β/°	95.790(4)	101.040(10)	72.344(9)
γ/°	90.00	90.00	83.028(8)
V/ Å^3	1462.45(60)	3028.37(594)	985.03(319)
Z	2	4	1
ρ/g.cm^{-3}	1.38814	1.50731	1.21296
μ/mm^{-1}	0.707	0.715	0.528
F(0 0 0)	636	1400	336
θ range/°	2.85-26.01	2.81-26.01	2.99-25.01
Reflections collected	5938	12106	6497
Completeness to θ	99.8%	99.8%	99.7%
Data/restraints/param.	2861/0/196	2976/0/214	3466/0/232
Goodness-of-fit on F^2	0.712	0.756	0.906
Final *R* indices [I>2$\sigma(I)^a$]	R_1 = 0.0434 wR_2 = 0.1066 wR_1 = 0.1066	R_1 = 0.0520 wR_2 = 0.1698	R_1 = 0.0844 wR_2 = 0.2145
λ/ Å	0.71073	0.71073	0.71073

[a] $R_1=\Sigma||F_0|-|F_c||/\Sigma|F_0|$ (based on reflections with $F_0^2>2\sigma F^2$). $wR_2=[\Sigma[w(F_0^2-F_c^2)^2]/\Sigma[w(F_0^2)^2]]^{1/2}$; $w=1/[\sigma^2(F_0^2)+(0.095P)^2]$; $P=[max(F_0^2, 0)+2F_c^2]/3$(also with $F_0^2>2\sigma F^2$).
